# Mix Design and Mechanical Properties of High-Performance Pervious Concrete

**DOI:** 10.3390/ma12162577

**Published:** 2019-08-13

**Authors:** Chao-Wei Tang, Chiu-Kuei Cheng, Ching-Yuan Tsai

**Affiliations:** 1 Department of Civil Engineering & Geomatics, Cheng Shiu University, No. 840, Chengching Rd., Niaosong District, Kaohsiung 83347, Taiwan; 2 Center for Environmental Toxin and Emerging-Contaminant Research, Cheng Shiu University, No. 840, Chengching Rd., Niaosong District, Kaohsiung 83347, Taiwan; 3 Super Micro Mass Research & Technology Center, Cheng Shiu University, No. 840, Chengching Rd., Niaosong District, Kaohsiung 83347, Taiwan; 4 Department of Agribusiness Management, National Pingtung University of Science & Technology, No. 1, Shuefu Rd., Neipu, Pingtung 91201, Taiwan

**Keywords:** high-performance pervious concrete, mix design, engineering properties

## Abstract

The mechanical properties of traditional pervious concrete are insufficient, which limits its application. In view of the imperfections of traditional permeable concrete in mechanics, this paper aimed to find a suitable material composition that can be used as a feasible mix design of high-performance pervious concrete, to essentially improve its mechanical properties. Based on the view that concrete is a two-phase material, in order to understand the rheological properties of the matrix, it was subjected to a rheological test, and then the filler aggregate was uniformly incorporated into the aforementioned matrix to further explore the composition and properties of the resulting pervious concrete. For the matrix, the orthogonal array employed was *L*_16_(4^5^), which consisted of five factors, each with four levels. Base on the fluidity and compressive strength of the tested matrix, three groups of suitable matrixes mix proportions were selected to serve as the matrix type for pervious concrete mix proportion design. Then, an orthogonal array *L*_9_(3^4^), which consisted of four controllable three-level factors, was adopted in the pervious concrete. The parameters investigated included the coarse aggregate size, fine aggregate content, matrix type, and aggregate-to-binder ratio. The test results demonstrate that the key factors affecting the compressive strength of the matrix and the pervious concrete were closely related to the cementitious material. In the matrix, the proportion of the cementitious material was the most important factor, while in the pervious concrete, the type of matrix was the most important factor.

## 1. Introduction

Most urban areas of the world are covered by reinforced concrete buildings and impervious roads, mainly due to the continuous development of contemporary urbanization [1]. Compared to natural soils, concrete pavements generally lack the ability to breathe, absorb heat, and infiltrate rainwater, leading to a series of environmental problems. As an example, nonpoint source pollution (NPS) is caused by surface runoff, rainfall, drainage, and seepage, which has been proven to be an important factor affecting the water quality of receiving waters [2]. Most of these runoffs are caused by impervious pavements in large parking lots, roads, and roofs. With the increasing population and urbanization of countries around the world, it is estimated that by 2050, the global urban population will have increased by more than two billion from the current level [3]. If this undue trend continues to develop, it will not only lead to the rapid expansion of the urban area but will also extend the corresponding area of surface impervious pavement [4]. To effectively increase the infiltration of rainwater at the surface to alleviate the drastic decline of the city’s groundwater level and other urban environmental problems such as NPS, pervious concrete has emerged.

Pervious concrete is a porous, lightweight concrete made from aggregates, hydraulic cement, and water, containing a large number of connected pores [4,5]. In pervious concrete, a carefully controlled amount of water and cementitious material is used to form a paste that forms a suitable coating around the aggregated particles and forms a highly permeable, interconnected void system that quickly drains water [4,5,6]. The literature shows that pervious concrete pavement is a unique and effective means of meeting the growing needs of the environment [6]. Compared to ordinary concrete pavements, the use of pervious concrete pavements in cities has many advantages [6,7,8,9,10,11,12,13,14,15,16,17,18,19,20,21,22,23,24,25,26], such as reducing stormwater runoff, reducing or eliminating detention tanks, reducing the impact and cost of rainwater treatment facilities, supplementing the groundwater level and underground aquifer, allowing more efficient land development, reducing surface pollutants, reducing the urban heat island effect, reducing flash floods, reducing water accumulation and backflow, preventing contaminated water from entering rivers to avoid affecting marine habitats, reducing dizziness caused by solar reflection, and supporting sustainable construction.

The proportion of pervious concrete varies from region to region. The standard pervious concrete used in the United States is a mixture of single-sized coarse aggregates and cement combined at a low water-to-cement ratio [25]. However, pervious concrete used in Europe and Japan is made with small-sized aggregates, sometimes with a small amount of fine aggregates [25]. The important hardening properties of typical pervious concrete, such as the density or unit weight, porosity, permeability, compressive strength, and flexural strength, are shown in Table 1 [6,11,25,26,27,28,29,30,31,32,33,34,35,36]. The unit weight of pervious concrete in the general field is approximately 1600–2000 kg/m^3^, which is already close to the upper limit of lightweight concrete [16]. In order to promote water permeation or flow in hardened concrete, there must be sufficient interconnected pores in the concrete, and the porosity should be at least 15%. Therefore, a generally pervious concrete mixture contains little or no sand to produce a large void content [6]. In general, the porosity of cast-in-place pervious concrete is 20% to 25%. However, a higher porosity will result in lower strength, whereas a lower porosity will result in higher strength [25]. According to the literature, if the porosity of pervious concrete is between 15% and 25%, its 28 day compressive strength can be greater than 13.8 MPa, and its permeability is about 3.4 mm/s [6,22,25,37]. From the point of view of sustainable development, replacing some of the cement with pozzolanic materials not only contributes significantly to energy conservation and carbon reduction but also improves the fresh properties and durability of pervious concrete materials [4,14,20,24,31,38,39,40]. It is worth noting that the use of fibers can also improve the low strength and low tensile resistance of pervious concrete [14,18,41,42,43,44,45].

Pervious concrete has been proven to have the potential to protect the environment and improve road safety. It has gradually become an important material for constructing parking lots and roads [7,8,9,10,11,12,13,25]. Compared with ordinary concrete, however, pervious concrete has low durability and strength and is mostly used in low traffic areas (such as parking lots, road shoulders, streets, and local roads). Therefore, to improve the existing shortcomings of pervious concrete and promote its further application, the present study aimed to determine a suitable material composition, as a feasible mix design of high-performance pervious concrete, to essentially improve its engineering properties. The parameters investigated included the coarse aggregate size, fine aggregate dosage, matrix type, and aggregate-to-binder ratio. Moreover, the effects of the experimental factors on the performance of the tested matrix and pervious concrete were evaluated by range analysis and variance analysis.

## 2. Experimental Details

### 2.1. Materials 

The materials used in the test included cement, silica fume, ultra-fine silica powder, coarse aggregates, fine aggregates, superplasticizers, a viscous agent, and polypropylene fiber. A locally produced Type I Portland cement with a specific gravity of 3.15 and a fineness of 3400 cm^2^/g was used. The silica fume was locally manufactured with a specific gravity of 2.75. Ultra-fine silica powder was purchased from abroad, and its specific gravity was 2.73. The fine aggregate was natural river sand with a specific gravity of 2.57, a water absorption of 1.45%, and a fineness modulus of 2.7. The coarse aggregate was gravel with a specific gravity of 2.59 and a water absorption of 1.30%. Superplasticizers and the viscous agent were local products (in accordance with the Chinese National Standards or the American Society for Testing Materials). The fibers were polypropylene fibers with a water absorption of 0% and a density of 0.9 g/cm^3^.

### 2.2. Test Variables and Experimental Design

In the matrix, the test parameters included the water–binder ratio, the relative proportions of cementitious materials (cement (C):Silica fume (SF):Ultra-fine silica powder (SFP)), the superplasticizer content, the viscous agent content, and the fiber content. As for pervious concrete, the test parameters included the coarse aggregate size, the fine aggregate content (the percentage of fine aggregates in the total aggregates), the matrix type, and the aggregate-to-binder ratio (A/B). Table 2 lists the levels of each test parameter of the matrix and its performance parameters, while Table 3 lists the levels of each test parameter of the concrete and its performance parameters. There are some disadvantages to considering all combinations of factors in a full-factorial design, such as the large number of experiments required, the long working hours, and the high cost [46]. In contrast, the Taguchi method utilizes orthogonal arrays for experimental design and concise analysis of variance and can be economically, operationally, and robustly based on only limited experimental data [47,48,49,50]. Therefore, the experimental scheme was designed using the Taguchi method. A typical Taguchi orthogonal array is named after *L_a_*(*b^c^*), which represents a total of *c* factors that can accommodate a maximum of *b* levels in *a* groups of experiments; that is, an experimental array has *a* rows and *c* columns. As shown in Table 4, an orthogonal array *L*_16_(4^5^) was adopted for the matrix, which consisted of five controllable four-level factors. Moreover, an orthogonal array *L*_9_(3^4^) was adopted for pervious concrete, which consisted of four controllable three-level factors (Table 5). Based on the test results of the fresh properties and compressive strength of the matrix, three groups of suitable matrix mix proportions were selected to serve as the matrix types for the pervious concrete mix proportion design. In Table 5, there are three types of matrix, CM1, CM2, and CM3, corresponding to M4, M1, and M7 in Table 4, respectively.

### 2.3. Mix Proportions and Casting of Specimens

According to the aforementioned orthogonal arrays, namely *L*_16_(4^5^) and *L*_9_(3^4^), the mix proportions of the matrix and the pervious concrete are listed in Table 6 and Table 7, respectively. The mixing of the matrix, cement, silica fume, ultra-fine silica powder, and fiber were first uniformly blended, and then water, superplasticizers, and the viscous agent were added. As for the pervious concrete, aggregates were cured indoors until the required saturated surface-dry condition was reached prior to mixing. The cementitious material, fine aggregates, and coarse aggregates were then evenly mixed, followed by the addition of water, superplasticizer, and the viscous agent. After thorough mixing, the fresh properties of these mixtures were measured and recorded. Afterwards, matrix specimens (50 by 50 by 50 mm cube) were cast from each mixture for the compressive strength test. In addition, the pervious concrete specimens required for each test were cast and compacted using an external vibrator. Along with each mix, cylindrical specimens (100 mm in diameter and 200 mm in height) were cast to determine the compressive strength and elastic modulus tests of concrete, cylindrical specimens (150 mm in diameter and 300 mm in height) were cast to determine the splitting strength test of concrete, and prism specimens (360 mm in length, 100 mm in width, and 100 mm in thickness) were cast to determine the flexural strength of concrete. After the specimens were cast, they were covered with wet hessian and polyethylene sheets overnight for 24 h. Demolding was then carried out, and each sample was placed in a laboratory water bath until the day before the mechanical test. Three representative samples were prepared per test for each mixture, and the average value was taken.

### 2.4. Test Methods and Data Analysis

The rheology of the matrix was tested using a viscometer (Brookfield HBDV-II + Mode, Middleboro, MA, USA) with a viscosity measurement accuracy of ±1% of the measurement range and a type spindle (#HB04) was selected. The fluidity test of the matrix was in accordance with ASTM C230 [51], and the compressive strength test was in accordance with ASTM C109 [52]. The unit weight of pervious concrete specimens was tested under air-dry conditions. The tests of void content and infiltration rate were performed according to ASTM C1688 [53] and ASTM C1701 [54], respectively. The void ratio of pervious concrete was determined by calculating the difference in weight between the oven dry sample and the saturated under water sample. The permeability of the pervious concrete samples was determined using a falling head permeability test apparatus. A 500 kN MTS servo controlled universal testing machine (MTS, Eden Prairie, MN, USA) with a viscosity measurement accuracy of ±1% of the measurement range was used for various mechanical tests. According to ASTM C39 specification [55], a compressive axial load was applied to the specimens at a continuous rate until failure occurred. The compressive strength was determined by dividing the maximum load by the cross-sectional area of the specimen. According to ASTM C469 [56], a series of compressive stress cycles up to approximately 40 percent of the measured compressive strength was applied. The modulus of elasticity of the specimen was corresponded to the average slope of the stress-strain responses captured during cyclic loading. Following the ASTM C496 [57], a diametric compressive load was applied along the length of the sample at a continuous rate until failure occurred. This loading induced tensile stresses on the plane containing the applied load, causing tensile failure of the specimen. The splitting tensile strength was determined by dividing the maximum applied load by the appropriate geometrical factors. In terms of flexural strength, ASTM C78 [58] was used to measure the flexural strength of concrete by using a simple beam with third-point loading. The specimen failed within the middle third of the span length in the tension area or underside of the specimen and the modulus of rupture was calculated.

In the Taguchi experimental design method, the deviation between the experimental value and the expected value is usually calculated by a so-called loss function. Traditionally, the value of the loss function is usually converted to the signal-to-noise ratio (*S*/*N*) ratio (*η*) [50]. In general, there are three categories of performance characteristics, as follows [50]:

(1) $$\text{The-smaller-the-better: }\eta =-10\times \log_{10}\left(MSD\right)=-10\times \log_{10}\left(\frac{1}{n}\sum_{i\;=\;1}^{n}y_{i}^{2}\right)$$

(2) $$\text{The-larger-the-better: }\eta =-10\times \log_{10}\left(MSD\right)=-10\times \log_{10}\left(\frac{1}{n}\sum_{i\;=\;1}^{n}\frac{1}{y_{i}^{2}}\right)$$

(3) $$\text{The nominal-the better: }\eta =-10\times \log_{10}\left(MSD\right)=-10\times \log_{10}\left(\frac{1}{n}\sum_{i\;=\;1}^{n}{\left(y_{i}-y_{0}\right)}^{2}\right)$$

where *MSD* is the mean squared deviation around the target value; *n* is the number of repetitions or observations; *y_i_* is the observed data; and *y*_0_ is the desired nominal value.

In this study, the observed flow values of the matrixes were set to a nominal level, while the observed values of the mechanical properties of the matrixes and pervious concretes were set to maximum levels. In addition, optimization of the observations was further detected by variance analysis. This was mainly achieved by separating the total variability of the *S*/*N* ratios into the contributions of each of the process parameters and the errors [59].

## 3. Results and Discussion

### 3.1. Test Results of Matrixes

#### 3.1.1. Rheological Behavior of Matrixes

Fresh matrix can be regarded as a thick suspension that is obtained by uniformly adding cement to water. Basically, the rheological behavior of this suspension structure is closely related to the water–binder ratio, the size and distribution of the binder particles, the interactions between the binder particles, and the ability of the binder particle surfaces to adsorb water molecules. The initial stage of matrix formation (in this case, a relatively thin solution), usually the cement particles, will show an agglutination phenomenon called flocculation, and then the flocculation tends to link into a continuous aggregate, this time called the flocculent state. When stirring the fresh matrix, the shear force generated by the stirring will break or destroy the original flocculation structure so that the previously aggregated particles can be uniformly dispersed in water, and this behavior is the cause of thixotropy. If the shear rate of the spindle is kept at a certain value, the matrix maintains an equilibrium structure and exhibits a distinct and invariable viscosity. When the shearing force ceases, the exact opposite of the above process occurs, i.e., the flocculation structure is formed again. Therefore, depending on the shear conditions applied, both opposing conditions may occur. In other words, when the shear force acts, the cement slurry exhibits a fluid behavior, and on standing, the flocculation structure makes the matrix comparable to a weak solid and has significant yield stress. In view of this, the deformation behavior of the matrix under stress should be essentially interpreted by the viscosity test results.

**Figure 1:**
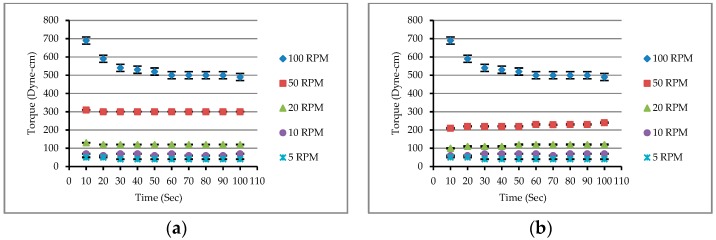
The relationship between torque and time of matrix (M13): (**a**) the spindle’s rotational speed under acceleration conditions; (**b**) the spindle’s rotational speed under deceleration conditions.

As is clear from Figure 1, the measured torque value of the spindle immersed in the matrix was initially high, regardless of the rising or falling branch of the torque versus time curve. Especially at 100 and 50 revolutions per minute (RPM), the torque value of the spindle was relatively large. When the spindle rotated for a period of time, the agglomeration structure around it was interrupted successively, resulting in a gradual decrease in torque, so the drop in the torque value was large. This situation was not obvious at low speeds (20, 10, and 5 RPM). In other words, the torque versus time curve at low speeds was fairly flat, as shown in Figure 1. This indicates that the measured torque data did not have a large drop and approached a stable value. The test results show that at high rotation speeds, the torque versus time curve took about 30 seconds to become gentler, i.e., to be in a more stable state.

**Figure 2:**
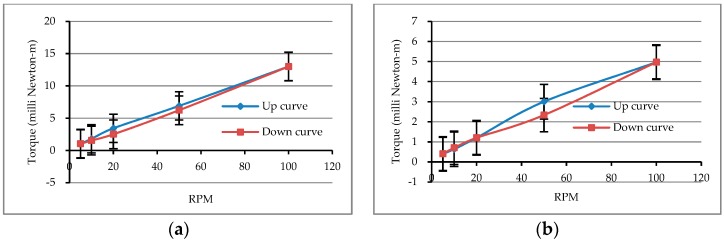
Hysteresis loop of matrixes: (**a**) M9; (**b**) M13.

In addition to the above, the hysteresis loop phenomenon also showed an effect on the rheological test results of the matrix, which was caused by the difference in the rotational speed of the spindle from high to low or from low to high. According to the test results, there were hysteresis loops in different matrixes, as shown in Figure 2, but fortunately, they were not large. Moreover, the relationship between torque and speed in the “down” curves was linear in comparison with that in the ”up” curves, indicating that the test method in the “down” curves was more in line with the rheological behavior measurement, making it more resilient. Taken together with the above test results and the literature [60], the speed of the experimental instrument should not be too small (about 0.06 RPM or less), because an insufficient rotational speed will not be able to break the agglomerate structure continuously, resulting in a high measured viscosity. Therefore, the rotational speed of the spindle used in this study was relatively high (5–100 RPM), and the speed was gradually decreased, that is, the change in rotational speed of the spindle was 100 → 50 → 20 → 10 → 5 RPM.

#### 3.1.2. Rheological Test Results of Matrixes

The results of the rheological tests on the matrixes are shown in Table 8. The mix proportions of the M1, M5, M9, and M13 are pure cement pastes. Pure cement pastes can be divided into two parts: The liquid phase (water) and the solid phase (cement particle). It can be seen from Table 8 that the main factor affecting the rheological behavior of the pure cement pastes is the water/cement ratio.

**Figure 3:**
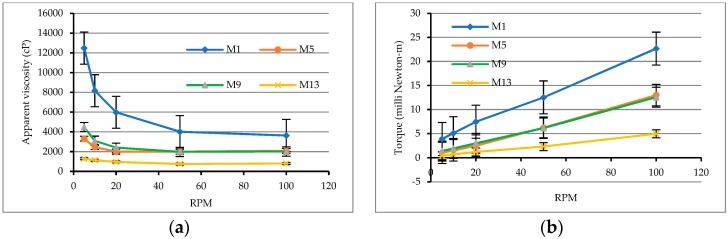
Rheological test results of pure cement pastes: (**a**) relationship between viscosity and spindle speed; (**b**) relationship between torque and spindle speed.

In addition, Figure 3a shows the correlation curve between the rotational speed of the spindle and the apparent viscosity of the pure cement pastes. As is clear from the figure, the apparent viscosity decreased as the water–cement ratio increased. This is mainly because the fresh cement pastes gradually became colloidal suspensions of solid particles of different sizes, and their structures were mainly controlled by the size distribution of the cement particles. When the water–cement ratio increased, the solid-phase particle content in the unit volume of the slurry decreased, and the interparticle distance became larger so that the attractive forces between the particles became smaller and the viscosity that could be possessed became relatively smaller. As can be seen from Figure 3b, under the same rotational speed conditions, the larger the water–cement ratio was, the smaller the rotational torque was, and there was a linear relationship between the torque (T) and rotational speed (N) of the spindle. This result shows that these matrixes had appropriate rheological behavior and confirmed that they complied with the Bingham model. Moreover, the y-intercept, g, and the slope, h, in the T–N curve can be obtained from Figure 3b. It can be found that the values of g and h decreased with an increase in the water–cement ratio, and the changing trend was quite obvious. Therefore, the rheological parameters can be used to evaluate the rheological properties of the matrix. The smaller the g value is, the easier the matrix to overcome the flow obstruction is; the smaller the value of h is, the smaller the viscosity of the matrix is.

**Figure 4:**
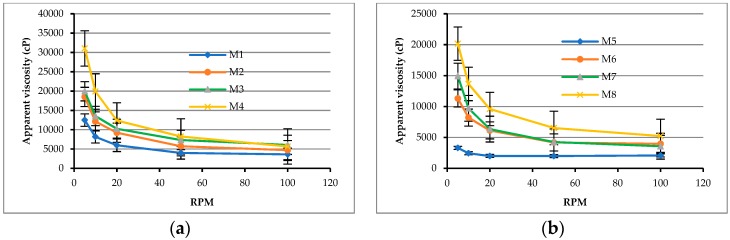

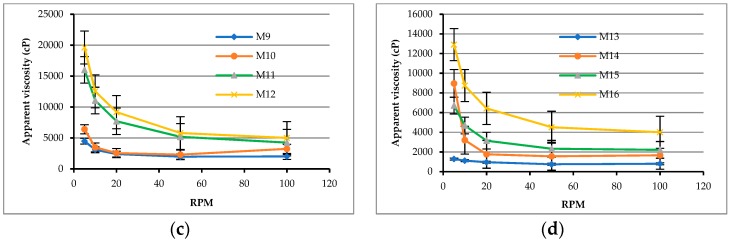
Relationship between the viscosity and the spindle speed of matrixes: (**a**) W/B = 0.28; (**b**) W/B = 0.31; (**c**) W/B = 0.34; (**d**) W/B = 0.37.

The rheological analysis results confirmed that the water–binder ratio is an important factor affecting the rheological properties of the cement paste. For example, in the case of a cement paste having a relatively high water–binder ratio, the water molecules can disperse the attraction between the cementitious particles and thus exhibit a low viscosity. With low water–binder ratios, where the number of bonds between the cementitious particles, the bonding strength, and the friction between the particles are relatively increased, an additional dispersant such as superplasticizers must be considered to obtain plasticity and flow. Therefore, this study used silica fume and ultra-fine silica powders to replace part of the cement. Each matrix was subjected to a rheological test under conditions in which the amounts of superplasticizer, viscous agent, and polypropylene fiber were changed. Figure 4 shows that as the amounts of silica fume and ultra-fine silica powder increased, the viscosities of the matrixes also changed. Taking matrixes with a water-to-binder ratio of 0.28 as an example (i.e., M1, M2, M3, and M4), when the amounts of viscous agent and polypropylene fiber increased, the apparent viscosities of the matrixes also increased significantly. In particular, at low speeds, the apparent viscosity of M4 was more than 30,000 cP.

#### 3.1.3. Flow Value and Compressive Strength of Matrixes

**Figure 5:**
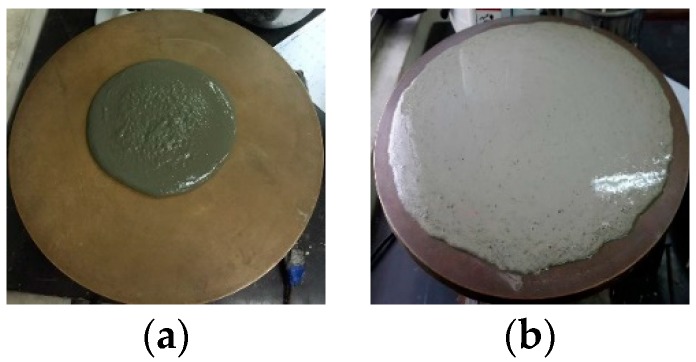
Flow state of matrixes. (**a**) M4; (**b**) M13.

Table 9 shows the experimental results of the matrixes and the corresponding *S*/*N* ratios using Equation (2). As shown in Table 9, the flow value of each matrix ranged from 43.7 to 265.5 mm. The M8 mix had the smallest flow value (43.7 mm), and the M13 mix had the largest flow value (265.5 mm), as shown in Figure 5. It can be seen from Table 9 that some of the matrixes were more viscous and their flow values were less than 60 mm; however, some of the matrixes had low viscosity values, and their flow values were greater than 200 mm. In order to avoid the phenomenon of the paste drain down in pervious concrete, the target value of the flow was set to 90 mm. The 7 day compressive strength of the matrixes ranged from 61.3 to 87.3 MPa. The water–binder ratio of M16 was 0.37, and its compressive strength was the lowest (61.3 MPa), while the M1 mix had a water-to-binder ratio of 0.28 and the highest compressive strength (87.3 MPa). Although Abram’s law (an empirical equation for the compressive strength of concrete as a function of its water-to-cement ratio) does not apply to permeable concrete, it still plays a key role. A higher water–binder ratio may make the matrix of the permeable concrete less viscous, and if subjected to slight vibrations, the slurry may fill the voids in the concrete; however, a lower water–binder ratio makes mixing difficult and the slurry cannot evenly coat the coarse aggregates and cannot fully exert its bonding effect, thereby reducing the strength of pervious concrete. If the amount of cementitious materials is determined, there is an optimal water–binder ratio that allows the slurry, such as a thin shell, to adhere evenly to the surfaces of the coarse aggregates so that the pervious concrete can achieve the maximum compressive strength.

Due to the characteristics of the selected orthogonal array, the effects of each test parameter at different levels can be evaluated separately. Taking the water–binder ratio as an example, the mean *S*/*N* ratio of the first level was calculated by averaging the *S*/*N* ratios of experiments M1–M4. The influence of each selected factor on the performance characteristic investigated is described in detail below.

**Figure 6:**
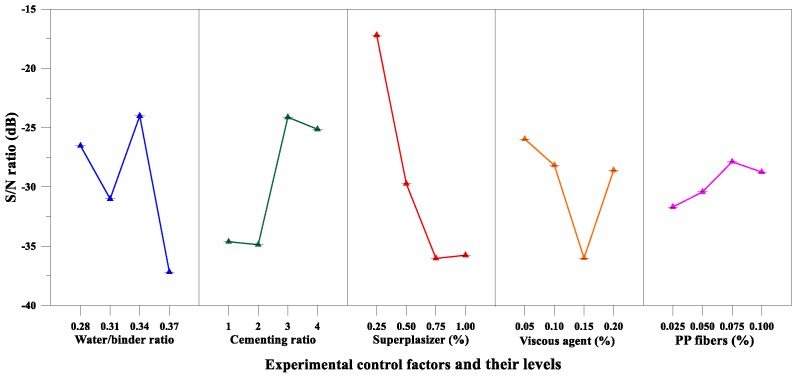
*S*/*N* response graph showing the flow values of matrixes.

Table 10 lists the mean *S*/*N* ratio at each parameter level for flow value, while Figure 6 shows the *S*/*N* response graph for flow value. The delta column in Table 10 shows the difference between the maximum and minimum values of the mean *S*/*N* ratio from Level 1 to Level 4. In theory, the importance can be evaluated by the delta value of each factor—the larger the delta value is, the greater the influence of the level of change of the factor on the performance parameter and the more important the factor is. As shown in Equation (3), the larger the *S*/*N* ratio is, the smaller the variance in flow value around the desired value is (i.e., the nominal—the better). From Table 10 and Figure 6, it can be seen that the superplasticizer content was the most important factor affecting the responses; the maximum response value occurred with the lowest superplasticizer content. However, the degree of influence of each factor on the test results cannot be estimated with precise quantities, and a criterion cannot be put forward to judge whether the examined factors are significant. In view of this, a statistical method, variance analysis, was used to further explore the test results. The results of the analysis of variance of the flow value are given in Table 11. In addition, the *F* values were obtained with a 95% level of confidence, and the percentage contribution of each parameter was also calculated. The superplasticizer content was the most significant factor that contributed to the target value of the flow of the matrix. The percentage contributions of these factors were as follows: superplasticizer content (53.37%), proportion of cementitious materials (18.78%), water–binder ratio (18.19%), and viscous agent content (9.65%). Thus, based on the results of the *S*/*N* ratio and variance analysis, the optimal combination of the experimental control factors for achieving nominal flow value is A_3_B_3_C_1_D_1_E_3_, i.e., water–binder ratio at level 3, proportion of cementitious materials at level 3, superplasticizer content at level 1, viscous agent content at level 1, and fiber content at level 3.

**Figure 7:**
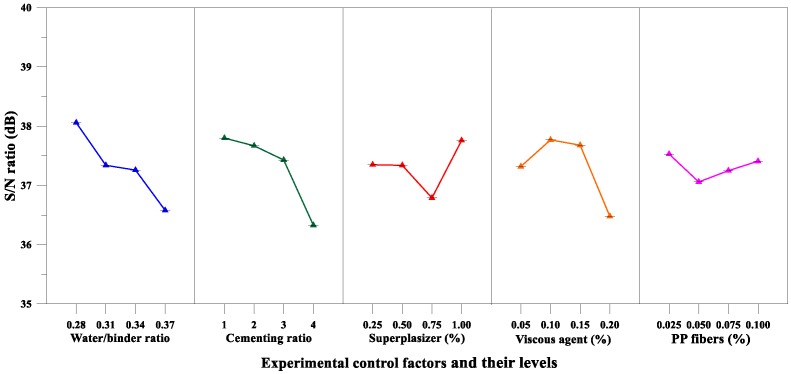
*S*/*N* response graph showing the compressive strength of the matrixes.

From Table 10 and Figure 7, it can be seen that the water–binder ratio and proportion of cementitious materials were the significant factors affecting compressive strength; the maximum response occurred at the lowest water–binder ratio. On the other hand, the results of the variance analysis for compressive strength are given in Table 11. The proportion of cementitious materials was the most significant factor contributing to the compressive strength of the matrix. The percentage contributions of these factors were as follows: Proportion of cementitious materials (30.04%), superplasticizer content (23.91%), water–binder ratio (23.69%), and viscous agent content (22.37%). Thus, based on the results of the *S*/*N* ratio and variance analysis, the optimal combination of the experimental control factors for achieving maximum compressive strength is A_1_B_1_C_4_D_2_E_1_.

### 3.2. Test Results of Pervious Concretes

The 28-day test results of the pervious concretes and the corresponding *S*/*N* ratios are shown in Table 12. As can be seen from Table 12, the unit weight of pervious concrete specimens was between 1507 and 1708 kg/m^3^, the void content of the pervious concrete was between 20.6% and 25.7%, and the hydraulic conductivity was between 21.6 and 24.2 mm/s. It can be seen from the composition of the pervious concrete that as the amount of fine aggregate increased, the void content tended to decrease. In addition, the unit weight was inversely proportional to the void content, while the hydraulic conductivity increased as the void content increased. Overall, the mechanical properties of the C3 mix are superior. 

#### 3.2.1. Compressive Strength

**Figure 8:**
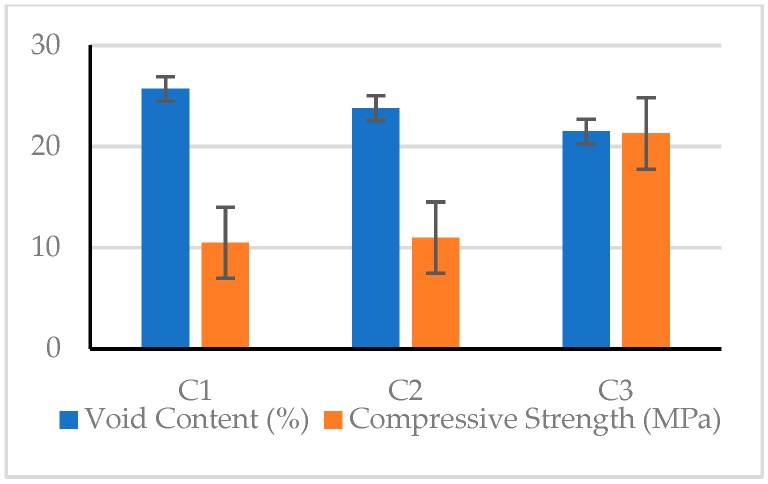
Relationship between compressive strength and void content.

It can be seen from Table 12 that the 28 day compressive strength of the pervious concrete was between 10.1 and 21.3 MPa. The C9 mix had the lowest compressive strength (10.1 MPa), and the C3 mix had the highest compressive strength (21.3 MPa). The difference between the compressive strengths of the various mixtures was very significant. Under the same conditions of coarse aggregate size, the compressive strength generally increased with the increase of the amount of fine aggregate content or void content. Taking C1–C3 mixtures with a coarse aggregate size between 4.75 and 9.5 mm as an example, the compressive strength was inversely proportional to the void content, as shown in Figure 8. This result shows that the compressive strength of pervious concrete is closely related to its mix proportions. 

**Figure 9:**
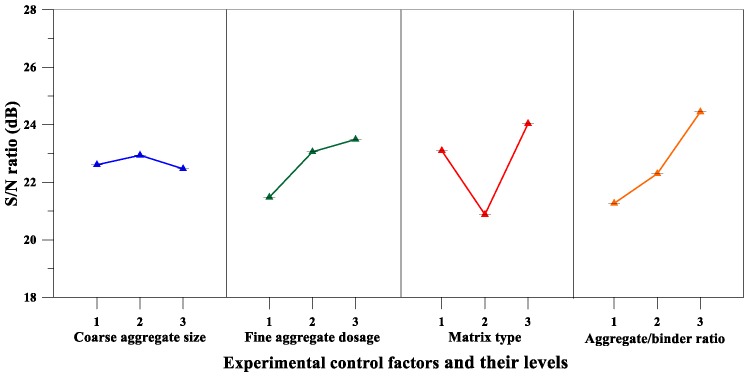
*S*/*N* response graph for the compressive strength of pervious concretes.

Moreover, Table 13 lists the mean *S*/*N* ratio at each level for the compressive strength parameters, whereas Figure 9 shows the *S*/*N* response graph for compressive strength. From the analysis results of Table 13 and Figure 9, it can be concluded that the aggregate-to-binder ratio was the significant factor affecting compressive strength; the maximum response occurred at an aggregate-to-binder ratio of 0.34. For the variance analysis results for compressive strength, the matrix type was the most important factor affecting the compressive strength of the pervious concrete, as shown in Table 14. The percentage contributions of these factors were as follows: Matrix type (43.54%), aggregate-to-binder ratio (39.99%), and fine aggregate content (16.47%). Therefore, based on the analysis results of the above range analysis and variance analysis, the optimal combination of the experimental control factors for achieving maximum compressive strength is A_2_B_3_C_3_D_3_.

#### 3.2.2. Elastic Modulus

**Figure 10:**
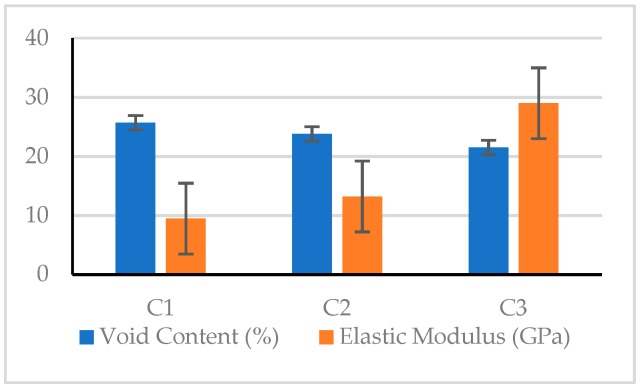
Relationship between elastic modulus and void content.

In terms of the elastic modulus, the test results showed values between 9479.2 and 29001.5 MPa. The C1 mix had the lowest elastic modulus (9479.2 MPa), and the C3 mix had the highest elastic modulus (29001.5 MPa). The maximum value was three times the minimum value, indicating a very significant difference between the elastic moduli of the various mixtures. In the case where the coarse aggregate had the same particle size, the elastic modulus generally decreased as the void content increased. In other words, the elastic modulus of the pervious concrete is closely related to its material composition. Taking the C1–C3 mixtures as an example, the elastic modulus was inversely proportional to the void content, as shown in Figure 10. 

**Figure 11:**
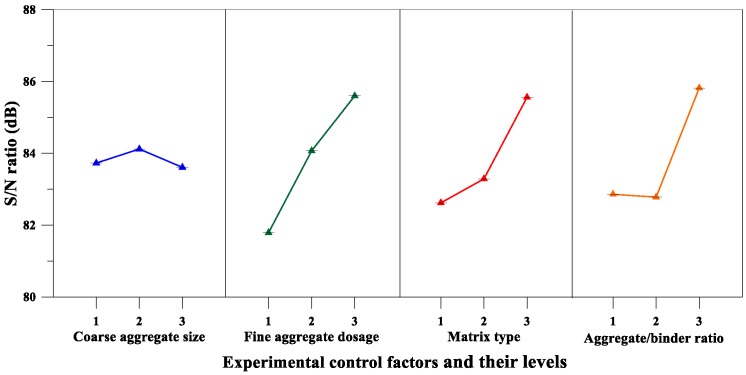
*S*/*N* response graph showing the elastic modulus of pervious concretes.

According to the average *S*/*N* ratio of the elastic modulus parameters of Table 13 and the elastic modulus *S*/*N* response diagram of Figure 11, the fine aggregate content was an important factor affecting the elastic modulus of pervious concrete, and the maximum response was at the third fine aggregate content level. Further, the results of the variance analysis for the elastic modulus are given in Table 14. It can also be confirmed from Table 14 that the fine aggregate content is the most important factor affecting the elastic modulus of the pervious concrete. The percentage contributions of these factors were as follows: Fine aggregate content (39.51%) and aggregate-to-binder ratio (32.16%). Therefore, based on the aforementioned analysis results, the optimal combination of the experimental control factors for achieving maximum elastic modulus is A_2_B_3_C_3_D_3_.

#### 3.2.3. Flexural Strength

**Figure 12:**
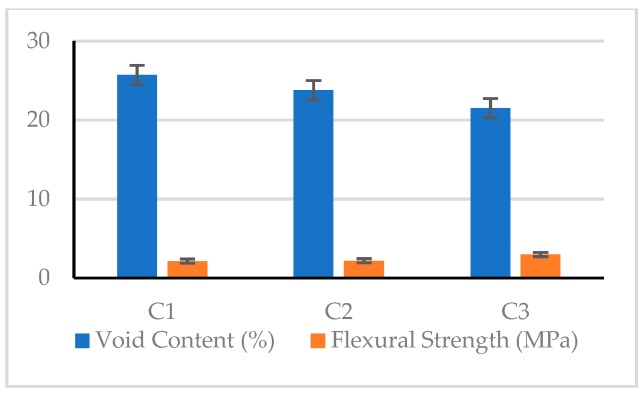
Relationship between flexural strength and void content.

As for the bending strength of the pervious concrete, the test results showed values between 1.88 and 2.99 MPa. The C4 mix had the lowest value (1.88 MPa), and the C3 mix had the highest value (2.99 MPa). There was a significant difference between the flexural strength of the various mixtures. In the case where the coarse aggregate had the same particle size, the flexural strength generally decreased as the void content increased. This shows that the flexural strength of the pervious concrete is closely related to its material composition. Taking the C1–C3 mixtures as an example, the flexural strength was inversely proportional to the void content, as shown in Figure 12. 

**Figure 13:**
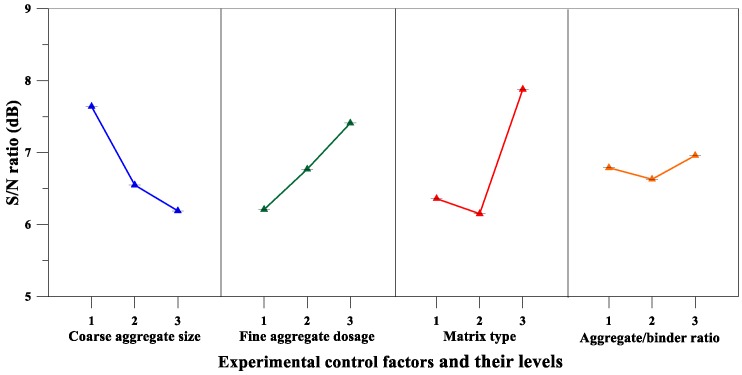
*S*/*N* response graph showing the flexural strength of pervious concretes.

On the other hand, as can be seen from Table 13 and Figure 13, the matrix type was an important factor affecting the flexural strength of the pervious concrete, and the maximum response was at the third level. Moreover, the results of the variance analysis for flexural strength in Table 14 also confirm that the matrix type was the most significant factor affecting the flexural strength of the pervious concrete. The percentage contributions of these factors were as follows: matrix type (52.54%), coarse aggregate size (29.38%), and fine aggregate content (18.08%). Summarizing the aforementioned analysis results, it can be inferred that the optimal combination of the experimental control factors for achieving maximum flexural strength is A_1_B_3_C_3_D_3_.

#### 3.2.4. Splitting Strength

**Figure 14:**
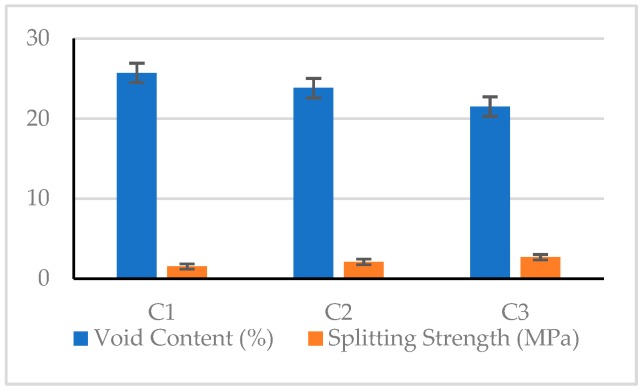
Relationship between splitting strength and void content.

Regarding the splitting strength of pervious concrete, the test results showed values between 1.43 and 2.69 MPa. The C4 mix had the lowest value (1.43 MPa), and the C3 had the highest value (2.69 MPa). The maximum value was 1.88 times the minimum value, indicating a very significant difference between the splitting strength of the various mixtures. In the case where the coarse aggregate had the same particle size, the splitting strength generally as the void content increased. In other words, the splitting strength of the pervious concrete is closely related to its material composition. Taking the C1–C3 mixtures as an example, the splitting strength was inversely proportional to the void content, as shown in Figure 14. 

**Figure 15:**
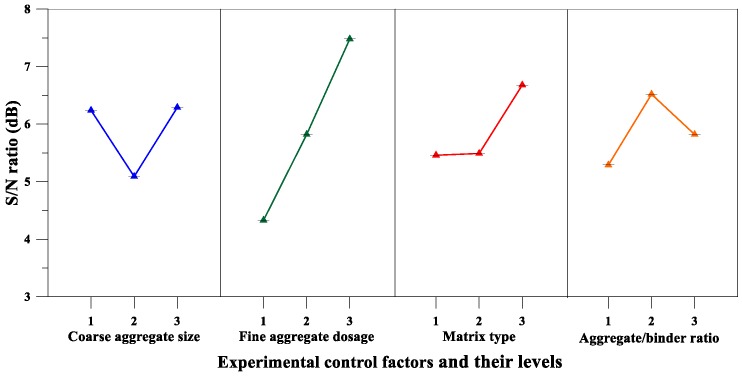
S/N response graph showing the splitting strength of pervious concretes.

On the other hand, the analysis results of Table 13 and Figure 15 confirm that the fine aggregate content was a significant factor affecting the splitting strength of the pervious concrete. Moreover, the results of the variance analysis for the splitting strength are given in Table 14, and the results also correspond to the fact that the fine aggregate content was the most significant factor affecting the splitting strength of pervious concrete. The percentage contributions of these factors were as follows: fine aggregate content (55.24%) and matrix type (42.71%). Summarizing the results of Table 13 and Table 14, it can be inferred that the optimal combination of the experimental control factors for achieving maximum splitting strength is A_3_B_3_C_3_D_2_.

#### 3.2.5. Confirmation Tests

To verify the optimal combination of the experimental control factors obtained using the Taguchi method, confirmation tests were carried out. Table 15 shows the results of the confirmation tests. The confirmation test results show that the optimal combination of the experimental control factors proposed by the Taguchi method can obtain the maximum test results for each performance parameter.

## 4. Conclusions

The results show that the key factors affecting the compressive strength of the matrix and the pervious concrete are closely related to the cementitious material. For the matrix, the superplasticizer content was shown to be the most important factor affecting the target value of flow, while the proportion of cementitious material was the most important factor affecting the compressive strength. Based on the fluidity and compressive strength of the tested matrix, three matrixes with suitable mix proportions were selected to serve as the matrix types for the mix proportion design of pervious concrete. The test results confirmed that the matrix type was the most significant factor affecting the compressive strength of the pervious concrete. The percentage contributions of each factor were as follows: matrix type (43.54%), aggregate-to-binder ratio (39.99%), and fine aggregate content (16.47%). Further, the matrix type was also the most significant factor affecting the flexural strength of the pervious concrete. On the other hand, the fine aggregate content was the most significant factor that contributed to the elastic modulus of the pervious concrete. Moreover, the fine aggregate content was also the most significant factor affecting the splitting strength of the pervious concrete. The percentage contributions of each factor were as follows: Fine aggregate content (55.24%) and matrix type (42.71%).

## Figures and Tables

**Table 1:** Properties of pervious concrete.

Void Ratio (%)	Unit Weight (kg/m^3^)	Permeability (mm/s)	28 day Compressive Strength (MPa)	Flexural Strength (MPa)	Reference
15–35	N/A *	N/A	N/A	2.5–3.9	[11]
19–34	1666–2034	10.2–15.0	13.5–25.2	N/A	[25]
19	N/A	N/A	26.0	4.4	[27]
20–30	1890–2083	NA	17.6–32.1	3.9–5.7	[28]
11–15	N/A	0.25–1.78	N/A	4.2–7.5	[29]
18–31	N/A	N/A	11.0–25.0	N/A	[30]
N/A	N/A	N/A	19.0	N/A	[12]
15–25	1602–2002	2.0–5.3	5.5–20.7	1.0–3.8	[31]
18–31	N/A	4.3–17.0	3.2–18.6	1.1–3.1	[6]
21–28	N/A	8.6–19.8	5.1–15.9	1.9–3.2	[32]
23–26	1890–1930	7.5–10.2	14.5–17.5	N/A	[33]
15–27	1766–1985	8.9–12.2	2.6–13.6	N/A	[34]
28–37	1750–1830	9.2–17.3	5.7–10.1	N/A	[35]
NA	1965–2067	3.8–6.1	8.5–17.2	1.1–4.8	[36]

Note: * N/A = not available.

**Table 2:** Parameters and design levels for matrix.

Parameter (Experimental Control Factor)	Levels of Parameter				Performance Parameter
	1	2	3	4	
Water–Binder Ratio, A	0.28	0.31	0.34	0.37	Flow value (mm) 7-day Compressive Strength (MPa)
Proportion of Cementitious Materials, B	PA *	PB	PC	PD	
Superplasticizer Content, C (%)	1.0	1.3	1.6	1.9	
Viscous Agent Content, D (%)	0.1	0.2	0.3	0.4	
Fiber Content, E (%)	0.025	0.050	0.075	0.100	

Notes: * PA = C (cement):SF (silica fume):SFP (ultra-fine silica powder) = 10:0:0; PB = C:SF:SFP = 9.6:0.2:0.2; PC = C:SF:SFP = 9.2:0.4:0.4; PD = C:SF:SFP = 8.8:0.6:0.6.

**Table 3:** Parameters and design levels for pervious concrete.

Parameter (Experimental Control Factor)	Levels of Parameter			Performance Parameter
	1	2	3	
Coarse Aggregate Size, A (mm)	4.75–9.5	9.5–12.5	12.5–19.0	Compressive Strength (MPa) Elastic Modulus (MPa) Flexural Strength (MPa) Splitting Strength (MPa)
Fine Aggregate Content, B (%)	0	5	10	
Matrix Type, C	CM1 *	CM2	CM3	
Aggregate to Binder Ratio, D	High	Medium	Low	

Notes: * There are three types of matrix, CM1, CM2, and CM3, corresponding to M4, M1, and M7 in Table 4, respectively.

**Table 4:** *L*_16_(4^5^) orthogonal array for matrix.

Experiment Number	Parameter (Level)				
	Water–Binder Ratio	Proportion of Cementitious Materials	Superplasticizers Content (%)	Viscous Agent Content (%)	Fiber Content (%)
M1	0.28 (1) *	PA (C:SF:SFP = 10:0:0) (1)	0.25 (1)	0.05 (1)	0.025 (1)
M2	0.28 (1)	PB (C:SF:SFP = 9.6:0.2:0.2) (2)	0.50 (2)	0.10 (2)	0.050 (2)
M3	0.28 (1)	PC (C:SF:SFP = 9.2:0.4:0.4) (3)	0.75 (3)	0.15 (3)	0.075 (3)
M4	0.28 (1)	PD (C:SF:SFP = 8.8:0.6:0.6) (4)	1.00 (4)	0.20 (4)	0.100 (4)
M5	0.31 (2)	PA (C:SF:SFP = 10:0:0) (1)	0.50 (2)	0.15 (3)	0.100 (4)
M6	0.31 (2)	PB (C:SF:SFP = 9.6:0.2:0.2) (2)	0.25 (1)	0.20 (4)	0.075 (3)
M7	0.31 (2)	PC (C:SF:SFP = 9.2:0.4:0.4) (3)	1.00 (4)	0.05 (1)	0.050 (2)
M8	0.31 (2)	PD (C:SF:SFP = 8.8:0.6:0.6) (4)	0.75 (3)	0.10 (2)	0.025 (1)
M9	0.34 (3)	PA (C:SF:SFP = 10:0:0) (1)	0.75 (3)	0.20 (4)	0.050 (2)
M10	0.34 (3)	PB (C:SF:SFP = 9.6:0.2:0.2) (2)	1.00 (4)	0.15 (3)	0.025 (1)
M11	0.34 (3)	PC (C:SF:SFP = 9.2:0.4:0.4) (3)	0.25 (1)	0.10 (2)	0.100 (4)
M12	0.34 (3)	PD (C:SF:SFP = 8.8:0.6:0.6) (4)	0.50 (2)	0.05 (1)	0.075 (3)
M13	0.37 (4)	PA (C:SF:SFP = 10:0:0) (1)	1.00 (4)	0.10 (2)	0.075 (3)
M14	0.37 (4)	PB (C:SF:SFP = 9.6:0.2:0.2) (2)	0.75 (3)	0.05 (1)	0.100 (4)
M15	0.37 (4)	PC (C:SF:SFP = 9.2:0.4:0.4) (3)	0.50 (2)	0.20 (4)	0.025 (1)
M16	0.37 (4)	PD (C:SF:SFP = 8.8:0.6:0.6) (4)	0.25 (1)	0.15 (3)	0.050 (2)

Note: * The numbers in parentheses indicate the level of the factor. The level of each factor has been explained in Table 2.

**Table 5:** *L*_9_(3^4^) orthogonal array for pervious concrete.

Experiment Number	Parameter (Level)			
	Coarse Aggregate Size (mm)	Fine Aggregate Content (%)	Matrix Type	Aggregate-to-Binder Ratio (weight ratio)
C1	4.75–9.5 (1)	0 (1)	CM1 (1)	6.2 (1)
C2	4.75–9.5 (1)	5 (2)	CM2 (2)	5.9 (2)
C3	4.75–9.5 (1)	10 (3)	CM3 (3)	5.6 (3)
C4	9.5–12.5 (2)	0 (1)	CM2 (2)	5.6 (3)
C5	9.5–12.5 (2)	5 (2)	CM3 (3)	6.2 (1)
C6	9.5–12.5 (2)	10 (3)	CM1 (1)	5.9 (2)
C7	12.5–19 (3)	0 (1)	CM3 (3)	5.9 (2)
C8	12.5–19 (3)	5 (2)	CM1 (1)	5.6 (3)
C9	12.5–19 (3)	10 (3)	CM2 (2)	6.2 (1)

Note: The numbers in parentheses indicate the level of the factor. The level of each factor has been explained in Table 3.

**Table 6:** Mix proportions of matrix.

Mix No.	W/B	W (kg/m^3^)	C (kg/m^3^)	SF (kg/m^3^)	UFS (kg/m^3^)	SP (kg/m^3^)	VA (kg/m^3^)	PP (kg/m^3^)
M1	0.28	450.24	1673.75	0.00	0.00	16.74	1.67	0.23
M2	0.28	442.18	1601.85	33.37	33.37	21.69	3.34	0.45
M3	0.28	434.07	1530.39	66.54	66.54	26.70	5.01	0.68
M4	0.28	425.99	1459.37	99.50	99.50	31.70	6.65	0.90
M5	0.31	467.45	1593.73	0.00	0.00	21.63	4.98	0.90
M6	0.31	469.65	1525.49	31.78	31.78	16.58	6.37	0.68
M7	0.31	459.29	1457.65	63.38	63.38	30.28	1.59	0.45
M8	0.31	461.14	1390.20	94.79	94.79	25.42	3.17	0.23
M9	0.34	485.47	1521.00	0.00	0.00	25.35	6.32	0.45
M10	0.34	481.11	1456.07	30.33	30.33	30.02	4.56	0.23
M11	0.34	496.01	1391.51	60.50	60.50	15.21	3.03	0.90
M12	0.34	512.10	1387.63	90.50	90.50	19.72	1.51	0.68
M13	0.37	506.34	1454.63	0.00	0.00	28.74	3.14	0.68
M14	0.37	510.22	1392.70	29.01	29.01	25.10	1.45	0.90
M15	0.37	510.62	1331.11	57.87	57.87	18.91	5.80	0.23
M16	0.37	536.42	1327.56	86.58	86.58	14.51	4.34	0.45

Notes: W/B = water–binder ratio; W = water; C = cement; SF = silica fume; USF = ultra-fine silica powder; SP = superplasticizers; VA = viscous agent; PP = polypropylene fiber.

**Table 7:** Mix proportions of pervious concrete.

Mix No.	W/B	W (kg/m^3^)	C (kg/m^3^)	SF (kg/m^3^)	UFS (kg/m^3^)	SP (kg/m^3^)	VA (kg/m^3^)	PP (kg/m^3^)	CA (kg/m^3^)	FA (kg/m^3^)
C1	0.28	51.76	177.32	11.96	11.96	3.85	0.81	0.11	1249.31	0.00
C2	0.28	59.24	220.23	0.00	0.00	2.20	0.22	0.03	1234.39	64.70
C3	0.31	69.38	220.19	9.57	9.57	4.57	0.24	0.07	1206.25	134.03
C4	0.28	60.35	224.36	0.00	0.00	2.24	0.22	0.03	1256.43	0.00
C5	0.31	61.82	196.18	8.53	8.53	4.08	0.21	0.06	1256.01	66.11
C6	0.28	60.25	206.42	14.07	14.07	4.48	0.94	0.13	1245.54	138.39
C7	0.31	62.57	198.59	8.63	8.63	4.13	0.22	0.06	1273.57	0.00
C8	0.28	61.03	209.08	14.26	14.26	4.54	0.95	0.13	1263.97	66.52
C9	0.28	61.42	228.34	0.00	0.00	2.28	0.23	0.03	1274.15	141.57

Notes: W/B = water–binder ratio; W = water; C = cement; SF = silica fume; USF = ultra-fine silica powder; SP = superplasticizers; VA = viscous agent; PP = polypropylene fiber; CA = coarse aggregate; FA = fine aggregate.

**Table 8:** Results of the apparent viscosity of matrixes.

Mix No.	W/B	Proportion of Cementitious Materials	Apparent Viscosity (centi-Poise, cP)				
			Rotational Speed of Spindle (RPM)				
			100	50	20	10	5
M1	0.28	C:SF:SFP = 10:0:0	3627	4011	5973	8160	12480
M2	0.28	C:SF:SFP = 9.6:0.2:0.2	4720	5717	9227	12147	18480
M3	0.28	C:SF:SFP = 9.2:0.4:0.4	6085	7328	10247	13547	19947
M4	0.28	C:SF:SFP = 8.8:0.6:0.6	5664	8256	12400	19893	31040
M5	0.31	C:SF:SFP = 10:0:0	2080	1995	2000	2453	3307
M6	0.31	C:SF:SFP = 9.6:0.2:0.2	3969	4192	6133	8213	11307
M7	0.31	C:SF:SFP = 9.2:0.4:0.4	3580	4267	6347	9707	14933
M8	0.31	C:SF:SFP = 8.8:0.6:0.6	5237	6528	9600	13653	20160
M9	0.34	C:SF:SFP = 10:0:0	2011	1973	2400	3093	4480
M10	0.34	C:SF:SFP = 9.6:0.2:0.2	3248	2304	2560	3467	6400
M11	0.34	C:SF:SFP = 9.2:0.4:0.4	4251	5184	7707	11053	16000
M12	0.34	C:SF:SFP = 8.8:0.6:0.6	5000	5792	9173	12533	19627
M13	0.37	C:SF:SFP = 10:0:0	795	747	960	1120	1280
M14	0.37	C:SF:SFP = 9.6:0.2:0.2	1653	1557	1760	3200	8960
M15	0.37	C:SF:SFP = 9.2:0.4:0.4	2213	2325	3147	4693	6720
M16	0.37	C:SF:SFP = 8.8:0.6:0.6	4000	4512	6427	8747	12907

**Table 9:** Experimental results and signal-to-noise (*S*/*N*) ratios of the matrixes.

Mix No.	Experimental Results		*S*/*N* Ratio (dB)	
	Flow Value (mm)	7-Day Compressive Strength (MPa)	Flow Value	7 Day Compressive Strength
M1	97.3	87.3	−17.27	38.82
M2	54.5	85.7	−31.00	38.66
M3	51.0	79.1	−31.82	37.96
M4	70.0	69.1	−26.02	36.79
M5	207.0	82.5	−41.36	38.33
M6	79.0	69.5	−20.83	36.84
M7	116.7	76.5	−28.53	37.67
M8	43.7	67.0	−33.31	36.52
M9	145.9	64.2	−34.95	36.15
M10	241.5	85.7	−43.61	38.66
M11	88.5	79.2	−3.52	37.97
M12	95.0	65.0	−13.98	36.26
M13	265.5	78.7	−44.89	37.92
M14	249.5	67.1	−44.06	36.53
M15	132.6	64.0	−32.59	36.12
M16	67.1	61.3	−27.20	35.75

**Table 10:** *S*/*N* response table of matrixes.

Performance Parameter	Parameter (Experimental Control Factor)	Mean *S*/*N* Ratio (*η*, Unit: dB)				Delta (Max. *η* − Min. *η*)	Rank
		Level 1	Level 2	Level 3	Level 4		
Flow Value	Water–Binder Ratio, A	−26.53	−31.01	−24.01	−37.18	13.167	2
	Proportion of Cementitious Materials, B	−34.62	−34.87	−24.12	−25.13	10.759	3
	Superplasticizer Content, C (%)	−17.20	−29.73	−36.03	−35.76	18.831	1
	Viscous Agent Content, D (%)	−25.96	−28.18	−36.00	−28.60	10.040	4
	Fiber Content, E (%)	−31.69	−30.42	−27.88	−28.74	3.815	5
Compressive Strength	Water–Binder Ratio, A	38.06	37.34	37.26	36.58	1.477	1
	Proportion of Cementitious Materials, B	37.80	37.67	37.43	36.33	1.475	2
	Superplasticizer Content, C (%)	37.35	37.34	36.79	37.76	0.968	4
	Viscous Agent Content, D (%)	37.32	37.77	37.68	36.48	1.293	3
	Fiber Content, E (%)	37.53	37.06	37.25	37.41	0.473	5

**Table 11:** Variance analysis and *F* test for matrixes.

Performance Parameter	Parameter (Experimental Control Factor)	Sum of Square (*SS_Z_*)	Degree of Freedom	Variance (*MS_Z_*)	*F* Value (*F_Z_*)	Percentage Contribution (*P_Z_*)
Flow Value	Water–Binder Ratio, A	400.27	3	133.42	11.46	18.19
	Proportion of Cementitious Materials, B	412.14	3	137.38	11.80	18.78
	Superplasticizer Content, C (%)	932.11	3	310.70	26.69	53.37
	Viscous Agent Content, D (%)	228.75	3	76.25	6.55	9.65
	Fiber Content, E (%)	34.92	3	11.64	1.00	0.00
	All Other/Error	34.92	3	11.64	–	–
	Total	2008.20	15	669.40	–	100
Compressive Strength	Water–Binder Ratio, A	4.37	3	1.46	8.68	23.69
	Proportion of Cementitious Materials, B	5.41	3	1.80	10.75	30.04
	Superplasticizer Content, C (%)	1.89	3	0.63	3.76	23.91
	Viscous Agent Content, D (%)	4.16	3	1.39	8.26	22.37
	Fiber Content, E (%)	0.50	3	0.17	1.00	0.00
	All Other/Error	0.50	3	0.17	–	–
	Total	16.34	15	5.45	–	100

**Table 12:** Experimental results and *S*/*N* ratios of pervious concretes.

Experiment Number	Unit Weight (kg/m^3^)	*V_c_* (%)	*k* (mm/s)	Experimental Results (MPa)				*S*/*N* Ratio (dB)			
				*f_c_*′	*E_c_*	*f_r_*	*f_s_*	*f_c_*′	*E_c_*	*f_r_*	*f_s_*
C1	1507	25.7	24.2	10.5	9479.2	2.14	1.53	20.42	79.54	6.61	3.69
C2	1581	23.8	23.1	11.0	13209.3	2.19	2.10	20.83	82.42	6.81	6.44
C3	1654	21.5	22.1	21.3	29001.5	2.99	2.69	26.57	89.25	9.51	8.60
C4	1544	24.8	23.6	12.2	15057.8	1.88	1.43	21.73	83.56	5.48	3.11
C5	1602	23.1	22.8	14.6	18082.9	2.40	1.83	23.29	85.15	7.60	5.25
C6	1684	21.0	21.8	15.5	15222.2	2.13	2.22	23.81	83.65	6.57	6.93
C7	1556	24.2	23.3	13.0	13002.6	2.12	2.04	22.28	82.28	6.53	6.19
C8	1635	22.3	22.4	17.9	17101.6	1.97	1.94	25.06	84.66	5.89	5.76
C9	1708	20.6	21.6	10.1	15662.2	2.03	2.22	20.09	83.90	6.15	6.93

Note: *V_c_* = void content; *k* = hydraulic conductivity; *f_c_*′ = compressive strength; *E_c_* = elastic modulus; *f_r_* = flexural strength; *f_s_* = splitting strength.

**Table 13:** *S*/*N* ratio response table of pervious concretes.

Performance Parameter	Parameter (Experimental Control Factor)	Mean *S*/*N* Ratio (*η*, Unit: dB)			Delta (Max. *η* − Min. *η*)	Rank
		Level 1	Level 2	Level 3		
Compressive Strength	Coarse Aggregate Size, A (mm)	22.61	22.94	22.47	0.466	4
	Fine Aggregate Content, B (%)	21.48	23.06	23.49	2.010	3
	Matrix Type, C	23.10	20.88	24.04	3.164	2
	Aggregate-to-Binder Ratio, D	21.27	22.30	24.45	3.185	1
Elastic Modulus	Coarse Aggregate Size, A (mm)	83.73	84.12	83.61	0.504	4
	Fine Aggregate Content, B (%)	81.79	84.07	85.60	3.808	1
	Matrix Type, C	82.62	83.29	85.56	2.943	3
	Aggregate to Binder Ratio, D	82.86	82.78	85.82	3.039	2
Flexural Strength	Coarse Aggregate Size, A (mm)	7.64	6.55	6.19	1.455	2
	Fine Aggregate Content, B (%)	6.21	6.77	7.41	1.204	3
	Matrix Type, C	6.36	6.15	7.88	1.734	1
	Aggregate-to-Binder Ratio, D	6.79	6.63	6.96	0.328	4
Splitting Strength	Coarse Aggregate Size, A (mm)	6.24	5.09	6.29	1.198	4
	Fine Aggregate Content, B (%)	4.33	5.82	7.48	3.152	1
	Matrix Type, C	5.46	5.49	6.68	1.220	3
	Aggregate-to-Binder Ratio, D	5.29	6.52	5.82	1.231	2

**Table 14:** Variance analysis and *F* test for pervious concretes.

Performance Parameter	Parameter (Experimental Control Factor)	Sum of Square (*SS_Z_*)	Degree of Freedom	Variance (*MS_Z_*)	*F* Value (*F_Z_*)	Percentage Contribution (*P_Z_*)
Compressive Strength	Coarse Aggregate Size, A (mm)	0.35	3	0.12	1.00	0.00
	Fine Aggregate Content, B (%)	6.72	3	2.24	19.42	16.47
	Matrix Type, C	15.82	3	5.27	45.68	43.54
	Aggregate-to-Binder Ratio, D	15.83	3	5.28	45.71	39.99
	All Other/Error	0.35	3	0.12	–	–
	Total	38.72	12	12.91	–	100.00
Elastic Modulus	Coarse Aggregate Size, A (mm)	0.42	3	0.14	1.00	0.00
	Fine Aggregate Content, B (%)	22.04	3	7.35	53.08	39.51
	Matrix Type, C	14.26	3	4.75	34.35	28.33
	Aggregate-to-Binder Ratio, D	18.02	3	6.01	43.39	32.16
	All Other/Error	0.42	3	0.14	–	–
	Total	54.73	12	18.24	–	100.00
Flexural Strength	Coarse Aggregate Size, A (mm)	3.44	3	1.15	21.35	29.38
	Fine Aggregate Content, B (%)	2.18	3	0.73	13.52	18.08
	Matrix Type, C	5.38	3	1.79	33.38	52.54
	Aggregate-to-Binder Ratio, D	0.16	3	0.05	1.00	0.00
	All Other/Error	0.16	3	0.05	–	–
	Total	11.16	12	3.72	–	100.00
Splitting Strength	Coarse Aggregate Size, A (mm)	2.76	3	0.92	1.21	2.06
	Fine Aggregate Content, B (%)	14.92	3	4.97	6.52	55.24
	Matrix Type, C	2.90	3	0.97	1.27	42.71
	Aggregate-to-Binder Ratio, D	2.29	3	0.76	1.00	0.00
	All Other/Error	2.29	3	0.76	–	–
	Total	22.86	12	7.62	–	100.00

**Table 15:** Results of the confirmation tests.

Performance Parameter	Initial Combination	Test Results (MPa)	Optimal Combination	Test Results (MPa)
Compressive Strength	A_1_B_3_C_3_D_3_	21.3	A_2_B_3_C_3_D_3_	22.1
Elastic Modulus	A_1_B_3_C_3_D_3_	29001.5	A_2_B_3_C_3_D_3_	29843.2
Flexural Strength	A_1_B_3_C_3_D_3_	2.99	A_1_B_3_C_3_D_3_	3.02
Splitting Strength	A_1_B_3_C_3_D_3_	2.69	A_3_B_3_C_3_D_2_	2.75
